# *In Cellulo* Bioorthogonal Catalysis by Encapsulated **AuPd** Nanoalloys: Overcoming Intracellular Deactivation

**DOI:** 10.1021/acs.nanolett.2c03593

**Published:** 2023-01-17

**Authors:** Belén Rubio-Ruiz, Ana M. Pérez-López, Laura Uson, M. Carmen Ortega-Liebana, Teresa Valero, Manuel Arruebo, Jose L. Hueso, Victor Sebastian, Jesus Santamaria, Asier Unciti-Broceta

**Affiliations:** † Edinburgh Cancer Research, Institute of Genetics and Cancer, University of Edinburgh, Crewe Road South, Edinburgh EH4 2XR, U.K.; ‡ Department of Medicinal and Organic Chemistry and Unit of Excellence in Chemistry Applied to Biomedicine and Environment, Faculty of Pharmacy, Campus Cartuja s/n, University of Granada, 18071 Granada, Spain; § GENYO, Pfizer/University of Granada/Andalusian Regional Government, PTS Granada, Avda. Ilustración 114, 18016 Granada, Spain; ∥ TU Berlin, Institut für Biotechnologie, Aufgang 17-1, Level 4, Raum 472, Gustav-Meyer-Allee 25, 13355 Berlin, Germany; ⊥ Instituto de Nanociencia y Materiales de Aragón (INMA), CSIC-Universidad de Zaragoza, 50009 Zaragoza, Spain; # Department of Chemical Engineering and Environmental Technologies, University of Zaragoza, 50018 Zaragoza, Spain; ∇ Networking Research Center on Bioengineering Biomaterials and Nanomedicine (CIBER- BBN), Instituto de Salud Carlos III, 28029 Madrid, Spain

**Keywords:** palladium, gold, nanoalloys, catalysis, bioorthogonal, nanoencapsulation

## Abstract

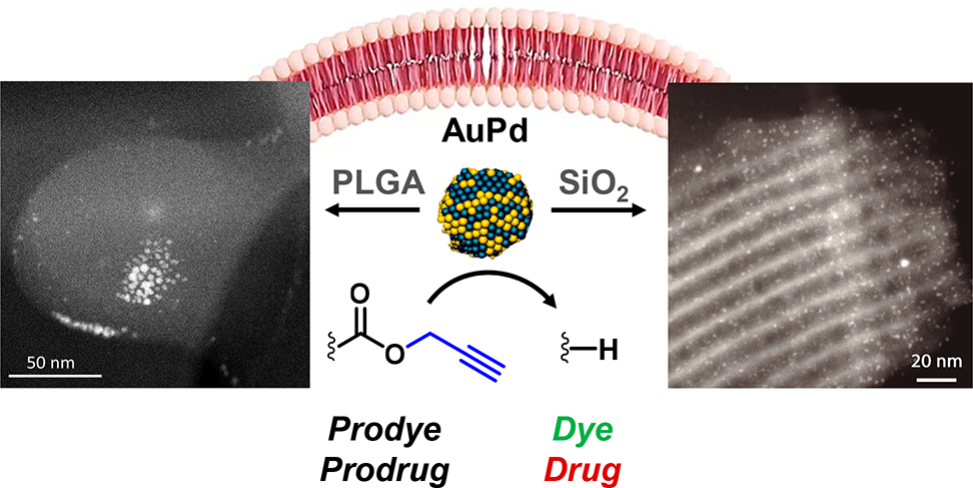

Bioorthogonal metallocatalysis has opened up a xenobiotic route to perform nonenzymatic catalytic transformations in living settings. Despite their promising features, most metals are deactivated inside cells by a myriad of reactive biomolecules, including biogenic thiols, thereby limiting the catalytic functioning of these abiotic reagents. Here we report the development of cytocompatible alloyed **AuPd** nanoparticles with the capacity to elicit bioorthogonal depropargylations with high efficiency in biological media. We also show that the intracellular catalytic performance of these nanoalloys is significantly enhanced by protecting them following two different encapsulation methods. Encapsulation in mesoporous silica nanorods resulted in augmented catalyst reactivity, whereas the use of a biodegradable PLGA matrix increased nanoalloy delivery across the cell membrane. The functional potential of encapsulated **AuPd** was demonstrated by releasing the potent chemotherapy drug paclitaxel inside cancer cells. Nanoalloy encapsulation provides a novel methodology to develop nanoreactors capable of mediating new-to-life reactions in cells.

In the early 2010s, the paths of bioorthogonal chemistry and nanotechnology crossed at the very center of the periodic table of elements,^1,2^ sparking the emergence of transition-metal catalysts (TMCs) as bioorthogonal nanotools.^3,4^ Over a decade later, numerous biocompatible TMC-based strategies with different features and functions have been developed for various biomedical applications, including *de novo* enzyme design,^5,6^ labeling/uncaging of biomolecules,^7,8^ or the release of metal-activated probes and therapeutics.^9,10^ Organometallic complexes,^11−15^ artificial metalloproteins,^5,6,16,17^ nanozymes and MOFs,^18−27^ metal-loaded exosomes and macrophages,^28−30^ and soft and hard micro/millidevices functionalized with metal nanoparticles (NPs)^10,31−36^ are representative examples of the wide diversity of catalytic systems reported to date.

The size and nature of the ligands or scaffolds bound to the metal atoms or NPs will dictate whether these nanoreactors are to exert their function in the extracellular or intracellular space, with each environment facing different conditions. While the interstitial liquid is mostly composed of water (up to 95%), intracellular bioorthogonal catalysis is severely restricted by biomolecule crowding (>20% protein by weight^37^) and the reductive environment of the cell cytoplasm. Under such stringent conditions, most TMCs are poisoned in relatively short periods of time. For this reason, technologies that protect abiotic metals from thiol-rich biomolecules and other reactive species and, at the same time, facilitate access of the substrate to metal active sites are in demand to overcome this issue.

A number of research laboratories have investigated the use of different TMCs (including noble metals such as **Ru**, **Pd**, **Au**, and **Pt**) and reactions (e.g., dealkylations, cross-couplings, cycloadditions, and ring-closure metathesis) in cells and organisms with the aim of expanding the chemical toolbox for bioorthogonal metallocatalysis. Depropargylations are one such reactions. *O*- and *N*-propargyl groups have been extensively used as a masking strategy in chemical biology and medicinal chemistry to render bioactive agents inactive, while activatable by abiotic metal catalysis.^10,21,28,32−35,38−44^ Because of the lack of natural “depropargylases”, this strategy offers superb control over bioorthogonal dissociative processes, enabling selective uncaging of drugs exclusively in the presence of a metal activator, even *in vivo*.^21,34,44^**Pd** and **Au** NPs stand out among the metallic nanocatalysts that catalyze depropargylation reactions. Interestingly, there has been an oversight of the reactivity of alloys of these or other noble metals so far. Encouraged by the potential benefits offered by alloyed nanomaterials,^45^ we embarked on a systematic study to investigate the capacity of a range of single-metal NPs and alloys to uncage a propargyl-masked prodye (**PocRho**)^10^ under physiological conditions.

Single-metal NPs of **Pd**, **Au**, **Pt**, and **Ru** and their corresponding bimetallic alloys were manufactured in a single step using tetrakis(hydroxymethyl)-phosphonium chloride as a simultaneous reducing agent and stabilizing ligand.^46−48^ (See the full experimental details in the Supporting Information.) NPs were then incubated with **PocRho**, a bis-propargyloxycarbonyl-protected prodye that releases green fluorescent rhodamine 110 (**Rho**) upon double depropargylation.^21^ Reactions were run at 37 °C in PBS in the absence and presence of serum to determine which metallic NPs were compatible not only with physiological media but also with supplements required in cell culture. An analysis of fluorescence intensity was carried out using a spectrofluorometer (PerkinElmer EnVision, λ_ex/em_ 480/535 nm). As shown in Figure 1a, single-metal **Pd** NPs and **Pd**-containing nanoalloys exhibited superior capabilities to uncage **Rho**, highlighting the performance of **AuPd** in serum-containing media. (See the kinetics study for best-performing NPs in Figure S1, Supporting Information.) Next, we tested the tolerability of human lung adenocarcinoma A549 cells to treatment with the most catalytic NPs identified in the fluorogenic study. Notably, **AuPd** nanoalloys induced no change in cell viability at any of the concentrations tested, whereas the rest of the NPs displayed variable levels of toxicity at 20 μg/mL (Figure 1b).

**Figure 1:**
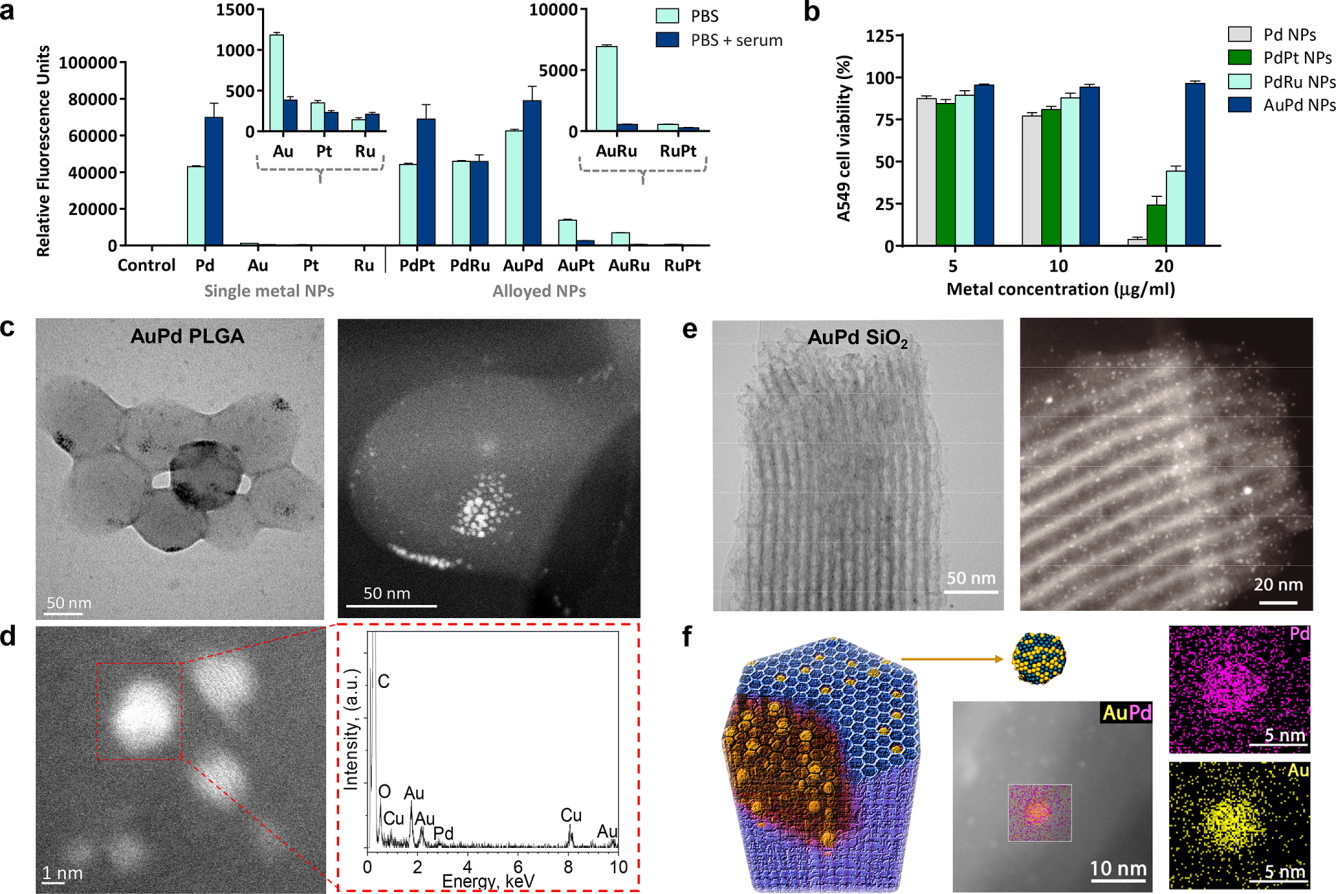
(a) Analysis of the conversion of nonfluorescent **PocRho** (100 μM) to fluorescent **Rho** after incubation with single-metal or alloyed NPs (20 μg metal/mL) in PBS and 10% FBS in PBS for 14 h. The fluorescence was measured at λ_ex/em_ 480/535 nm. Error bars: ±SD, *n* = 3. (b) A549 cell viability study after treatment with **Pd**, **PdPt**, **PdRu**, and **AuPd** NPs over a range of concentrations. Cell viability measured at day 5 using PrestoBlue reagent. Error bars: ±SD, *n* = 3. (c) Representative TEM (left) and HAADF-STEM (right) images of **AuPd PLGA** at different magnifications. (d) Elemental analysis of an **AuPd** NP embedded in **PLGA** by energy-dispersive X-ray spectroscopy (EDS). Analysis was carried out in the marked area of the HAADF-STEM image. (e) Representative TEM (left) and HAADF-STEM (right) images of **AuPd SiO**_**2**_ at different magnifications. (f) Schematic display of a **SiO**_**2**_ scaffold decorated with **AuPd** NPs and elemental mapping analysis of an **AuPd** NP by STEM-EDS (scanning mode).

Encouraged by the catalytic properties and tolerability of **AuPd**, we performed a preliminary *in cellulo* study to test the bioorthogonal reactivity of the NPs in A549 cells. Disappointingly, cancer cells pretreated with **AuPd** were not able to convert an inactive precursor of paclitaxel into its toxic form, indicating that these NPs are either unable to enter cells or rapidly deactivated in the cell cytoplasm. The latter is not surprising as metallic **Au** has a high affinity for thiol groups,^32^ which are ubiquitous in proteins and can bind to **Au** surfaces, sterically hindering the access of the substrate to the catalytic sites. Consequently, we decided to investigate different nanoencapsulation methods with the aim of enhancing nanoalloy delivery and protecting the metal NPs from direct contact with thiol-rich biomolecules in the crowded cell cytoplasm. Two kinds of materials were tested to encapsulate **AuPd**: poly(lactic-*co*-glycolic acid) (**PLGA**) and mesoporous silica nanorods (**SiO**_**2**_). Direct encapsulation of preformed **AuPd** NPs in the PLGA emulsion results in an inconsistent nanoalloy load distribution,^49^ making this method inadequate for our goals. Therefore, **AuPd-PLGA** was prepared by the water/oil/water emulsion and solvent evaporation method using a new methodology inspired by a recently reported procedure designed to produce **Pd** nanosheets *in situ* in PLGA.^50^ (See Figure S2 and experimental details in the Supporting Information.) Images from transmission electron microscopy (TEM and HAADF-STEM) clearly show 2 to 3 nm **AuPd** NPs embedded inside PLGA nanodrops of approximately 100 nm in diameter (Figure 1c,d and Figure S3). As shown in Figure 1c, the **AuPd** NPs were selectively loaded in each nanomatrix of PLGA, which is evidence of the efficiency of the loading method. Elemental analysis confirmed the alloyed composition of the **AuPd** NPs (Figure 1d), which demonstrates the versatility of the *in situ* approach to yield not only single-metal NPs^48^ but also **AuPd** nanoalloys, being the first procedure that facilitates the crystallization of alloyed NPs in double emulsions of PLGA. On the other hand, ordered mesoporous **SiO_2_** nanorods were prepared according to previously reported procedures,^52,53^ followed by amine-grafting with 3-aminopropyl-triethoxysilane and loading of preformed **AuPd** NPs. (See full experimental details in the Supporting Information.) TEM and elemental analysis show **AuPd** NPs embedded within the mesoporous nanorods (Figure 1e,f and Figures S4–S6). The contents of **Au** and **Pd** in the nanoencapulated NPs were measured by microwave plasma atomic emission spectrometer (MP-AES) to enable comparative studies using equivalent bimetal concentrations.

The **PocRho** fluorogenic test was then used to compare the catalytic efficacy of uncoated **AuPd** NPs versus nanoencapsulated **AuPd** at 37 °C in PBS with and without serum. Analysis revealed that **AuPd SiO**_**2**_ mediated the highest fluorescence signal, being superior to uncoated **AuPd** nanoalloys (Figure 2). This may be associated with the homogeneous dispersion of **AuPd** NPs throughout the pore channels of the scaffold.^51^ In contrast, **AuPd PLGA** demonstrated a significantly lower depropargylation capacity at equal metal concentrations. Subsequently, we studied the capacity of each of the materials to deliver **AuPd** into A549 cells. Two methods were used to study nanoalloy transcellular delivery: TEM, to visualize the metal presence in the cell cytoplasm, and inductively coupled plasma-atomic emission spectrometry (ICP-OES), for quantitative metal content analysis. Cells were treated with nanoencapsulated **AuPd** NPs for 30 min. After the media containing NPs were removed and the adhered cells were washed twice with PBS, cells were detached by trypsinization and centrifuged to further eliminate extracellular NPs by discarding the supernatant. Cells were then incubated on a coverslip for an additional 24 h, fixed (paraformaldehyde), and processed for TEM analysis. Rather than being adsorbed on the cell membrane, images verified the presence of **AuPd PLGA** and **AuPd SiO**_**2**_ in the cytoplasm of A549 cells, which appeared as separate NPs of various sizes across the cell cytoplasm (Figure 3a,b; see additional images in Figure S7, Supporting Information). On the other hand, an intracellular metal content study was carried out by incubating A549 cells with freestanding **AuPd** NPs, **AuPd PLGA**, and **AuPd SiO**_**2**_ (20 μg/mL of metal content), followed by the same procedure described above to remove extracellular NPs and thus ensure that only internalized nanoalloys were measured. Cell pellets were digested (10% HNO_3_) and analyzed by ICP-OES. As shown in Figure 3c, the content of **Au** and **Pd** in cells treated with uncoated **AuPd** was relatively low, which may in part explain the lack of capacity of these NPs to activate prodrugs inside cells. Notably, encapsulated **AuPd** achieved far superior intracellular delivery, highlighting the performance of **AuPd PLGA**, whose capacity to deliver the nanoalloys into A549 cells was approximately 25% higher than that of **AuPd SiO**_**2**_. As expected, the Au/Pd content ratio remained constant for the three nanocomposites.

**Figure 2:**
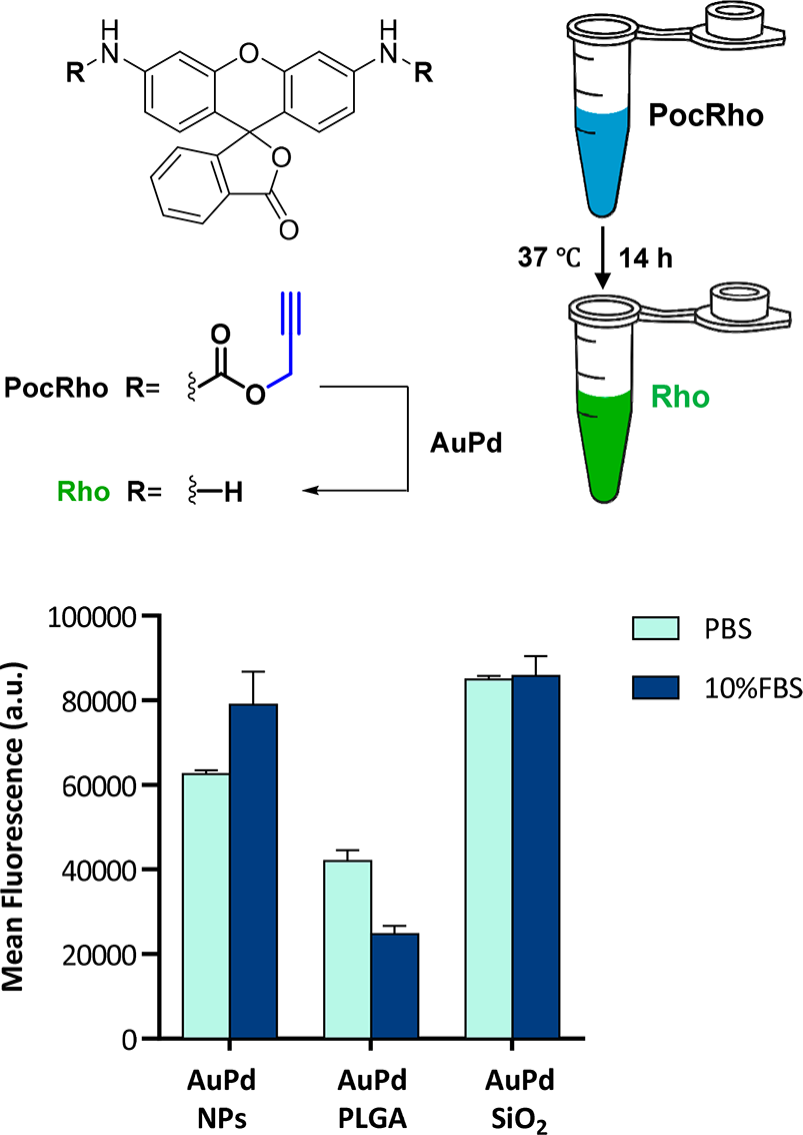
Analysis of the conversion of **PocRho** (100 μM) into **Rho** after incubation with naked **AuPd** NPs, **AuPd PLGA**, and **AuPd SiO**_**2**_ (20 μg metal/mL) in PBS and 10% FBS in PBS for 14 h. Fluorescence was measured at λ_ex/em_ 480/535 nm. Error bars: ±SD, *n* = 3.

**Figure 3:**
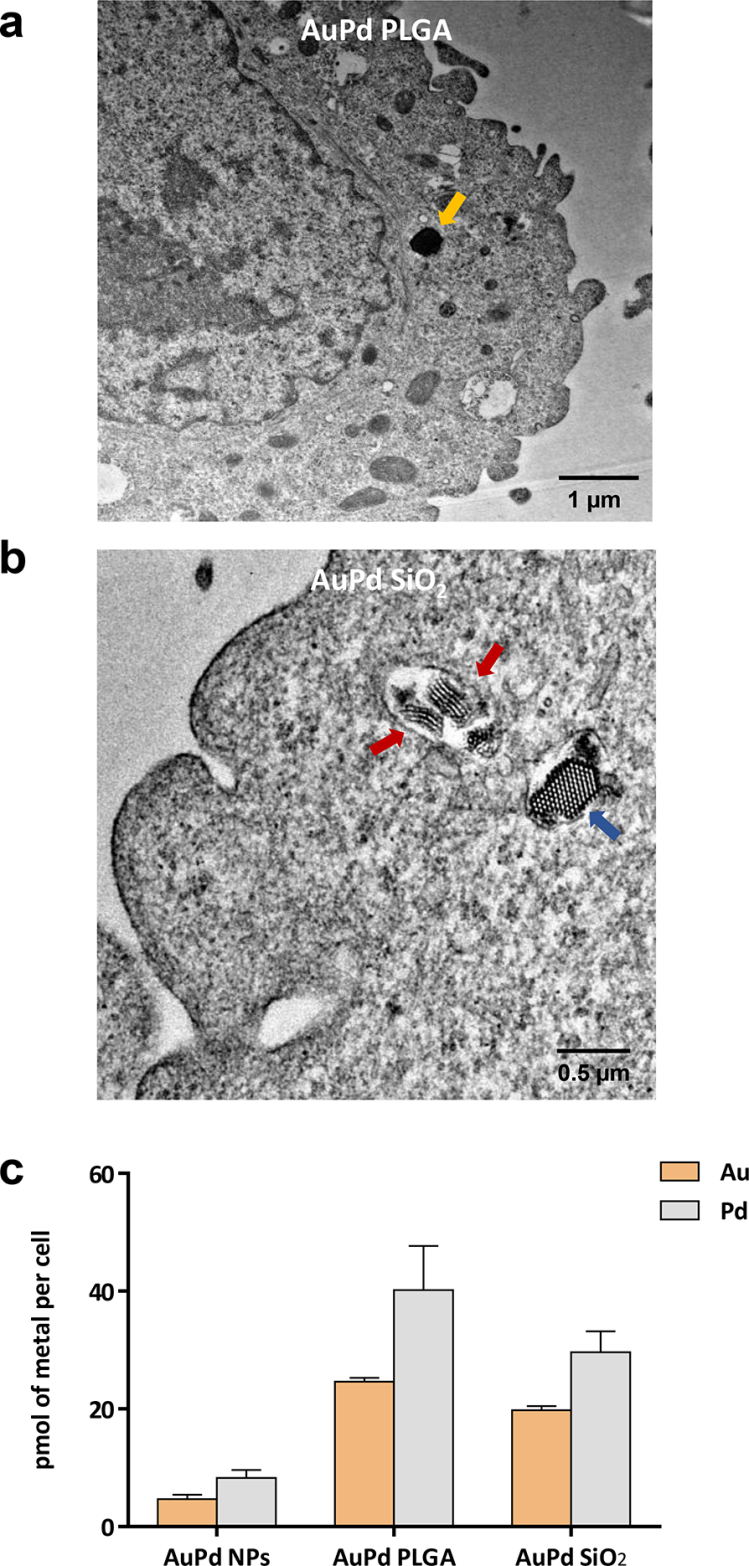
Nanoalloy internalization studies in lung cancer A549 cells. (a) Representative TEM image of the ultrathin cross-section of a cell treated with **AuPd PLGA**, showing the membrane and the cytoplasm. A NP is indicated with an orange arrow. Scale bar = 1 μm. (b) Representative TEM image of the ultrathin cross-section of a cell treated with **AuPd SiO**_**2**_, showing the membrane and the cytoplasm. Two laterally imaged NPs are indicated with red arrows. A transversally imaged NP is indicated with a blue arrow. Scale bar = 0.5 μm. (c) Quantification of Au and Pd content inside the cell (pmol of metal/cell). Analysis performed by ICP-OES. Error bars: ±SD, *n* = 3.

Encouraged by the improved catalytic properties of **AuPd SiO**_**2**_ in biological media and the superior transcellular carrier abilities of **AuPd PLGA**, an intracellular prodrug activation study was performed using the inactive chemotherapy precursor **Pro-PTX** (Figure 4a), which upon a single depropargylation reaction triggers a self-immolation cascade that releases highly toxic **PTX** (mechanism described in a previous work^35^). A549 cells were preincubated with either freestanding or encapsulated **AuPd** NPs (20 μg of metal/mL) for 30 min and then washed twice with PBS to eliminate extracellular nanoalloys. Vehicle (0.1% v/v DMSO) or **Pro-PTX** (1 μM) was added to the cells and incubated for 5 days. Cell viability was measured using the PrestoBlue assay. **PTX** (1 μM) treatment and untreated cells (0.1% v/v DMSO) were used as positive and negative controls, respectively. Cells treated with **Pro-PTX** (1 μM) in the absence of **AuPd** NPs were used as an additional negative control. As shown in Figure 4b, A549 cell proliferation was unaffected by treatment with either **Pro-PTX** or the **AuPd** nanoalloys. In contrast, the incubation of **Pro-PTX** with cells pretreated with encapsulated **AuPd** elicited highly potent inhibition of cancer cell proliferation, showing anticancer activity comparable to the direct treatment with **PTX**. Remarkably, the intracellular drug uncaging capacities of both encapsulation methods were essentially equivalent. This indicates that the superior delivery properties of **AuPd PLGA** compensates for its lower catalytic properties relative to those of **AuPd SiO**_**2**_. Of note, incubation of **Pro-PTX** with cells pretreated with “naked” **AuPd** NPs did not show any reduction of cell viability, in agreement with their reduced cellular internalization (as shown in Figure 3c). This study demonstrates that the encapsulation of metal nanoalloys can serve to improve both catalytic and cell delivery properties, thereby enabling the performance of intracellular bioorthogonal reactions.

**Figure 4:**
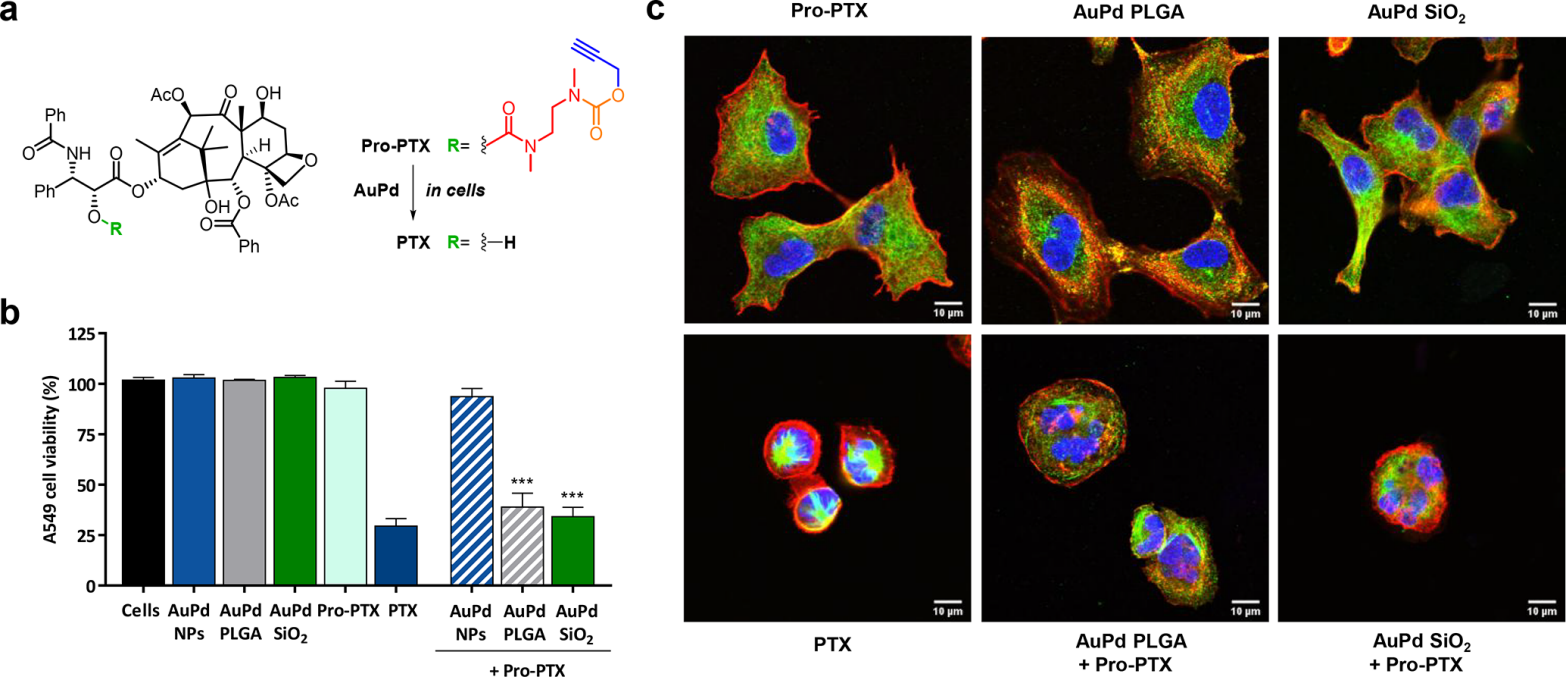
(a) **AuPd**-mediated conversion of **Pro-PTX** to **PTX**. (b) Intracellular prodrug activation assay in A549 lung cancer cells. Cells were treated with uncoated and encapsulated **AuPd** for 30 min prior to prodrug addition. Cell viability was measured at day 5. Experiments: 0.1% DMSO (untreated control); **AuPd** (20 μg metal/mL, −ve control); **Pro-PTX** (1 μM, −ve control); **PTX** (1 μM, +ve control); and **AuPd** + 1 μM **Pro-PTX** (activation assays). Error bars: ±SD, *n* = 3. Significance was determined by one-way analysis of variance (ANOVA): ***P < 0.001. (c) Immunofluorescence study of the microtubule distribution in A549 cells labeled with **AuPd PLGA** or **AuPd SiO**_**2**_ and treated with **Pro-PTX** (1 μM). Cells were incubated with encapsulated **AuPd** for 30 min prior to prodrug addition. Negative controls: **Pro-PTX (**1 μM), **AuPd PLGA**, and **AuPd SiO**_**2**_. Positive control: **PTX** (1 μM). 48 h after treatment, cells were fixed and stained for microtubules (green), actin filaments (red), and cell nuclei (blue). The panel shows the merged image of the three channels as maximal projections. Scale bar = 10 μm.

Finally, to validate that the combined treatment of encapsulated **AuPd** NPs and **Pro-PTX** results in the same antiproliferative mode of action as for the parent drug **PTX**, we studied microtubule stabilization by immunofluorescence.^54^ Cells were treated as previously described; fixed after 2 days of treatment; incubated with DAPI (cell nuclei stain), anti-α-tubulin IgG (for microtubules), and TRITC-phalloidin (for actin filaments); and imaged by confocal microscopy (Olympus FV1000). As shown in Figure 4c, negative controls did not induce changes in cell morphology. In contrast, treating A549 cells with **PTX** led to microtubule accumulation (green channel), round-shaped cells, and fragmented nuclei (independent channel images are shown in Figure S8, Supporting Information). Notably, equivalent morphological changes were observed in cells treated with both **Pro-PTX** and encapsulated **AuPd** NPs, evidence that the anticancer effect mediated by these combinations is the result of the intracellular generation of **PTX**.

In conclusion, we have studied the capacity of noble metal NPs (**Pd**, **Au**, **Pt**, and **Ru**, and their corresponding bimetallic alloys) to mediate depropargylation reactions in biological media and discovered that **AuPd** nanoalloys display superior catalytic properties and tolerability compared to any other single-metal or alloyed NP tested in this work. The enhanced catalysis elicited by alloyed **AuPd** NPs is likely a consequence of a combination of factors, including geometric and electronic effects on the NP surface^55^ and the fact that each metal mediates depropargylation reactions by different but complementary mechanisms.^32,38^ Regrettably, these bimetallic NPs show negligible bioorthogonal reactivity inside cells. To improve their *in cellulo* performance, we investigated two nanoencapsulation methods, both of which successfully enabled **AuPd**-mediated intracellular uncaging of the clinically approved drug **PTX**. Notably, each material promoted **AuPd** catalysis by different means: nondegradable mesoporous silica nanorods augmented the catalytic performance of the nanoalloys, whereas biodegradable **PLGA** matrixes enhanced transcellular NP delivery. By protecting **AuPd** NPs with scaffolds with distinct features, this investigation provides a novel and versatile strategy for protecting metal NPs and performing intracellular biorthogonal catalysis toward different applications, including the selective uncaging of probes and drugs inside cells.

## Supporting Information Available

Preparation and characterization of NPs, prodye, and prodrug; methods and experimental procedures; biological assays; and Figures S1–S8 (PDF)

## Abbreviations

DAPI: 4′,6-diamidino-2-phenylindole
DMEM: Dulbecco’s modified Eagle’s medium
DMSO: dimethyl sulfoxide
EDS: energy-dispersive X-ray spectroscopy
HAADF-STEM: high-angle annular dark-field scanning transmission electron microscopy
ICP-OES: inductively coupled plasma atomic emission spectroscopy
IgG: immunoglobulin G
MP-AES: microwave plasma atomic emission spectrometer
MS: mass spectrometry
NP: nanoparticle
PBS: phosphate-buffered saline
PLGA: poly(lactic-co-glycolic acid
ppm: parts per million
r.t.: room temperature
SD: standard deviation
TEM: transmission electron microscopy
TMC: transition-metal catalyst
