# Supplementary material for: In Cellulo Bioorthogonal Catalysis by Encapsulated AuPd Nanoalloys: Overcoming Intracellular Deactivation
Source: Nano Lett. 2023 Jan 17;23(3):804–11. doi: 10.1021/acs.nanolett.2c03593 (PMC9912372; doi:10.1021/acs.nanolett.2c03593)
Supplement: Supplementary file 1 — nl2c03593_si_001.pdf [file nl2c03593_si_001.pdf]

SUPPORTING INFORMATION

In Cellulo Bioorthogonal Catalysis by Encapsulated AuPd Nanoalloys. Overcoming Intracellular Deactivation

Belén Rubio-Ruiz,[a,b,c,‡] Ana M. Pérez-López,[a,d,‡] Laura Uson,[e,f,‡] M. Carmen Ortega-Liebana,[a,b,c] Teresa Valero,[a,b,c] Manuel Arruebo,[e,f,g] Jose L. Hueso,[e,f,g,*] Victor Sebastian,[e,f,g,*] Jesus Santamaria,[e,f,g,*] and Asier Unciti-Broceta[a,*]

[a] Edinburgh Cancer Research, IGC, University of Edinburgh, Crewe Road South, Edinburgh EH4 2XR, UK.

[b] Department of Medicinal and Organic Chemistry and Unit of Excellence in Chemistry Applied to Biomedicine and Environment, Faculty of Pharmacy, Campus Cartuja s/n, University of Granada, 18071 Granada, Spain.

[c] GENYO, Pfizer/University of Granada/Andalusian Regional Government, PTS Granada, Avda. Ilustración 114, 18016 Granada, Spain.

[d] TU Berlin, Institut für Biotechnologie, Aufgang 17-1, Level 4, Raum 472, Gustav-Meyer-Allee 25, 13355 Berlin, Germany

[e] Instituto de Nanociencia y Materiales de Aragón (INMA), CSIC-Universidad de Zaragoza, 50009 Zaragoza, Spain.

[f] Department of Chemical Engineering and Environmental Technologies, University of Zaragoza, 50018 Zaragoza, Spain.

[g] Networking Research Center on Bioengineering Biomaterials and Nanomedicine (CIBER- BBN), Instituto de Salud Carlos III, 28029 Madrid, Spain.

Table of Contents

1. General 2

2. Synthetic and Characterization of Prodye, Prodrug and NPs 2

2.1 Synthesis of PocRho 2

2.2 Synthesis of Pro-PTX 2

2.3 Synthesis of single metal and alloyed NPs 3

3. Fluorogenic Studies 3

4. Synthesis and Characterization of Encapsulated AuPd NPs 4

4.1 Synthesis of AuPd PLGA 4

4.2 Synthesis of rod-shaped SBA-15 mesoporous supports (SiO_2) 5

4.3 Amine-grafting of mesoporous supports and bimetallic NPs loading 5

4.4 Characterization 5

5. Biological Studies 8

5.1 Cell culture 8

5.2 Tolerability study 8

5.3 Determination of AuPd PLGA and AuPd SiO_2 cellular uptake by TEM 8

5.4 Determination of AuPd PLGA and AuPd SiO_2 cellular uptake by ICP-OES 10

5.5 *In cellulo* Pro-PTX activation 10

5.6 Immunofluorescence study 10

6. References 12

1. General. Chemicals and solvents were purchased from Fisher Scientific, Sigma-Aldrich, VWR International Ltd., Merk Millipore and Evonik Industries. NMR spectra were recorded at ambient temperature on a 500 MHz Bruker Avance III spectrometer. High Resolution Mass Spectrometry was measured in a Bruker MicroTOF II. R_f values were determined on Merck TLC Silica gel 60 F254 plates under a 254 nm UV source. Purification was carried out by flash column chromatography using commercially available silica gel (220-440 mesh, Sigma-Aldrich).

2. Synthetic and Characterization of Prodye, Prodrug and NPs

2.1. Synthesis of PocRho. PocRho (bis-*N,N'*-propargyloxycarbonyl-rhodamine 110) was synthesized following a previously reported procedure [1]. Briefly, rhodamine 110 chloride was dissolved in dry DMF under nitrogen atmosphere. Propargyl chloroformate and triethylamine were then added dropwise to the mixture. The reaction mixture was stirred at room temperature for 48 h and purified by flash chromatography (hexane/ethyl acetate 2:1). Purity was >95% measured by TLC and NMR. **^{1}H NMR** (500 MHz, DMSO-d_6) δ 7.82 (d, J = 8.9 Hz, 1H), 7.55 (d, J = 9.8 Hz, 1H), 7.18 (d, J = 2.7 Hz, 1H), 7.11 (dd, J = 8.9, 2.7 Hz, 1H), 6.80 (dd, J = 9.8, 2.1 Hz, 1H), 6.30 (d, J = 2.0 Hz, 1H), 5.01 (d, J = 2.4 Hz, 2H), 3.70 (t, J = 2.4 Hz, 1H). **HRMS (ESI)** m/z $[M + H]^+$ calcd for $C_{15}H_{10}N_3O_1$, 252.06552; found, 252.06554. The spectral data matched the values reported in the literature [1].

2.2. Synthesis of Pro-PTX. Prodrug **Pro-PTX** was synthesized following a previously reported procedure [2]. 2′-(4-Nitrophenoxycarbonyl)paclitaxel was dissolved in dry DMF (1 mL) under a N_2 atmosphere and cooled down to 0 °C. *N*-(Propargyloxycarbonyl)-*N,N′*-dimethylethylenediamine (7 mg, 42 μmol) and DIPEA (12 μL, 70 μmol) were dissolved in dry DMF (0.5 mL) and added dropwise to the solution and the mixture was allowed to reach room temperature and stirred overnight. The reaction mixture was stirred for 18 h and purified by flash chromatography (DCM/MeOH 10:0.25). **^{1}H NMR** (500 MHz, DMSO-d6) δ 9.17 (d, J = 8.9 Hz, 1H), 7.98 – 7.94 (m, 2H), 7.87 – 7.81 (m, 2H), 7.73 (t, J = 7.5 Hz, 1H), 7.65 (t, J = 7.5 Hz, 2H), 7.59 – 7.53 (m, 1H), 7.49 (t, J = 7.3 Hz, 2H), 7.47 – 7.43 (m, 4H), 7.19 – 7.17 (m, 1H), 6.29 (s, 1H), 5.87 – 5.81 (m, 1H), 5.66 – 5.54 (m, 1H), 5.41 (d, J = 7.2 Hz, 1H), 5.30 – 5.12 (m, 1H), 4.88 (dd, J = 14.5, 8.5 Hz, 2H), 4.62 – 4.52 (m, 2H), 4.13 – 4.08 (m, 1H), 4.03 – 3.97 (m, 2H), 3.58 (d, J = 7.1 Hz, 1H), 3.46 (d, J = 6.6 Hz, 1H), 3.40 (s, 1H), 2.88 (s, 1H), 2.85 (s, 1H), 2.81 (s, 1H), 2.77 (d, J = 6.9 Hz, 1H), 2.72 (d, J = 0.6 Hz, 2H), 2.35 – 2.20 (m, 4H), 2.10 (d, J = 1.5 Hz, 3H), 1.81 – 1.78 (m, 4H), 1.62 (t, J = 13.0 Hz, 2H), 1.49 (s, 4H), 1.34 (s, 1H), 1.22 (s, 2H), 1.01 (d, J = 7.4 Hz, 6H). **HRMS (ESI)** m/z $[M + H]^+$ calcd for $C_{56}H_{64}N_3O_{17}$, 1050.42302; found, 1050.42118. The spectral data matched the values reported in the literature [2].

2.3. Synthesis of single metal and alloyed NPs. The synthesis of single and alloyed metal NPs was performed according to previous procedures [3-5]. The metal precursor (Pt, Au, Ru and Pd, see Table S1) was added to 15 mL of distilled water in a glass vial under magnetic stirring. Meanwhile, 333 µL of a 65 mM tetrakis(hydroxymethyl)phosphonium chloride (THPC) solution was added. After several minutes under stirring to promote molecular mixing, 165 µL of a 1 M NaOH solution was added to the glass vial. The reaction mixture was kept at room temperature for 4 d, wrapping an aluminum foil to preserve the reaction mixture from photothermal decomposition.

Table S1. Metal precursors and weight/volume of reagents considered in the synthesis of pure metallic NPs.

Pt	100 µL of Chloroplatinic(IV) acid hydrate 8wt% ($HPtClO_4$)
Au	300 µL of Gold(III) chloride hydrate 30 mM ($HAuCl_4$)
Ru	4.3 mg of ruthenium(III) chloride ($RuCl_3$)
Pd	6.7 mg of potassium tetrachloropalladate(II) (K_2PdCl_4)

Alloyed NPs were synthesized following the same procedure that the one described for single metal NPs, but it was considered the simultaneous addition of both metal precursors with 1:1 molar ratio (see Table S2). Regarding NPs that contain Pd, the stabilizing role of THPC was reinforced by the addition of 75 mg of polyvinylpyrrolidone (PVP, MW = 10.000 Da) before THPC addition.

Table S2. Metal precursors and weight/volume of reagents considered in the synthesis of alloyed metallic NPs.

RuPt	2.1 mg of Ru precursor + 50 µL of Pt precursor
AuPt	150 µL of Au precursor + 50 µL of Pt precursor
PdPt	50 µL of Pt precursor + 3.1 mg of Pd precursor
AuPd	150 µL of Au precursor + 3.1 mg of Pd precursor
PdRu	2.1 mg of Ru precursor + 3.1 mg of Pd precursor

3. Fluorogenic Studies

PocRho (100 µM) was dissolved in PBS or 10% FBS in PBS (1 mL) with uncoated and encapsulated **AuPd** NPs (20 µg metal / mL). The resulting mixtures were shaken at 1,200 rpm and 37°C in a Thermomixer for 14 h. Reaction crudes were centrifuged (13,000 rpm) for 5 min to sediment the NPs and avoid fluorescence quenching. Fluorescence intensity of supernatants were measured in a PerkinElmer EnVision 2101 multilabel reader ($\lambda_{ex/em}$= 480/535 nm). Experiments were performed in triplicates.

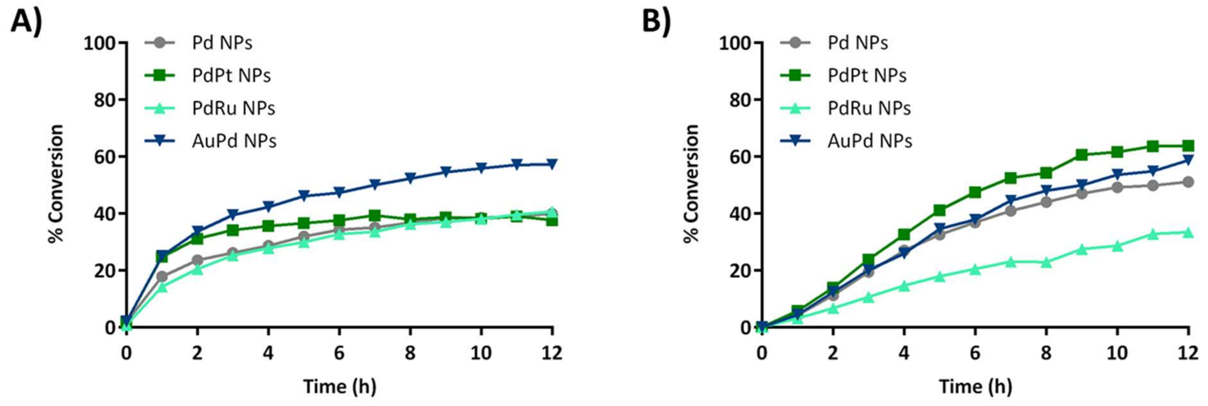

Figure S1. Kinetic study of the conversion of non-fluorescent **PocRho** (100 µM) into fluorescent **Rho** after incubation with single Pd and Pd-alloyed NPs (20 µg metal / mL) in PBS (A) and 10% FBS in PBS (B). Fluorescence measured at λex/em 480/535 nm. Conversion rates were calculated from **Rho** standard curve.

4. Synthesis and Characterization of Encapsulated AuPd NPs

4.1. Synthesis of AuPd PLGA. AuPd PLGA were prepared by modification of a previous procedure [6]. According to the modified method, 50 mg of PLGA polymer were dissolved into 1 mL of ethyl acetate and emulsified with 25 µL of Pd precursor solution and 25 µL of Au precursor solution (inner water phase). The Pd precursor solution was prepared by adding 8.9 mg of K_2PdCl_4 in 200 µL of water and Au precursor solution was prepared by adding 10 mg of $HAuCl_4$ in 700 µl of water. THPC was added to the metal solutions, preserving the same THPC/metal precursor molar ratio than in the production of alloyed metal NPs. The resulting mixture was sonicated in an ice bath for 15 s with a sonicator using a probe of 0.13 in. in diameter and 30% of amplitude. Then, the formed w/o emulsion was emulsified with 2 mL of 10% w/v sodium cholate solution at 30% amplitude for 15 s to obtain the w/o/w emulsion. 10 mL of 0.3% w/v sodium cholate solution were also added to promote the stability of the emulsion. The emulsion was introduced immediately after its formation in a CO-pressurized reactor and was kept at 30 °C for 40 min. The pressure of the autoclave was set at 6 bar. CO was solubilized and diffused through the aqueous and organic compartments of the emulsion and finally enabled the controlled reduction of metallic ions into atoms. The reduction of ions was easily observed by the hue change, since the emulsion turned into a dark color after the CO reduction process (see Figure S1). Finally, the double emulsion containing **AuPd NPs** was kept under continuous stirring at 600 rpm, for 3 h to evaporate the ethyl acetate. The evaporation of ethyl acetate promoted the PLGA crystallization into a nanoparticle size. The resulting NPs were washed by centrifugation, three times, at 7500 rpm for 10 min. Washed NPs were dispersed in 2 mL of distilled water, obtaining a 11 mg/mL solution.

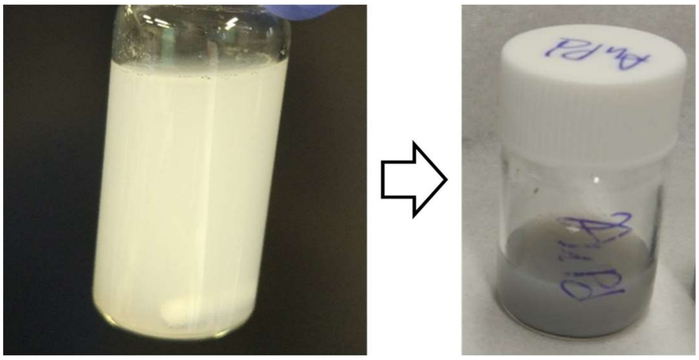

Figure S2. Representative images of the w/o/w emulsion loaded with **Au-Pd** precursors: Left (before reduction) and right (after CO reduction).

4.2. Synthesis of rod-shaped SBA-15 mesoporous supports (SiO_2). Ordered mesoporous silica nanorods were prepared according to previously reported procedures [7-9]. In brief, 1.2 g of Pluronic® P-123 and 0.014 g of NH_4F were first dissolved at 20 °C in 40 mL of HCl (1.75 M) solution under stirring. Upon complete dissolution, 2.75 mL of tetraethylorthosilicate (TEOS) and 8.5 mL heptane were added drop-wise and left under stirring for 8 min. The aged precursor mixture was hydrothermally heated at 100 °C for 24 h in a Teflon-lined autoclave. The solid product was then filtered, washed three times with distilled water, and dried at 60 °C overnight. Finally, the remaining surfactant was removed following a calcination protocol in a muffle furnace at 550 °C for 8 h, keeping a heating slope rate of 1 °C min^{-1}.

4.3. Amine-grafting of mesoporous supports and AuPd NPs loading. The resulting mesoporous rods were post-grafted with amine terminal groups with the aid of 3-aminopropyl-triethoxysilane (APTES). In a typical synthesis, 250 mg of SBA-15 calcined nanorods were dispersed in 18 mL of anhydrous toluene and kept stirring at 120 °C under reflux conditions in an argon ambient. After 2 h, 0.2 mL of APTES were added and kept under reflux for 8 h. The functionalized solid was filtered, washed 3 times with ethanol absolute and dried overnight at 85 °C. To complete the entrapment of preformed **AuPd** NPs, 20 mg of SBA-15 NRs-NH_2 were dispersed in 10 mL of the corresponding aqueous suspension containing **AuPd** and stirred for 2 h at room temperature. The solid was centrifuged twice and redispersed in 2.5 mL H_2O. Following this protocol, typical metal loadings of ca. 2% wt. were obtained.

4.4. Characterization. Au and **Pd** contents were measured on a Agilent 4100 MP-AES instrument after dissolving the sample in a mixture 5:1 of H_2O:aqua regia. Transmission electron microscopy (TEM) was performed using a FEI TECNAI T20 microscope operated at 200 keV. Samples were prepared by dropcasting 5 μL of the nanoparticle suspension on a holey carbon TEM grid. Aberration-corrected scanning transmission electron microscopy (Cs-corrected STEM) images were acquired using a high angle annular dark field detector (HAADF) in a FEI XFEG TITAN electron microscope operated at 200–300 kV. Elemental analysis was carried out with an EDAX detector in scanning mode. Samples were prepared by depositing 4 μL of the NP suspension on a holey carbon TEM grid. See Figures S2-S5.

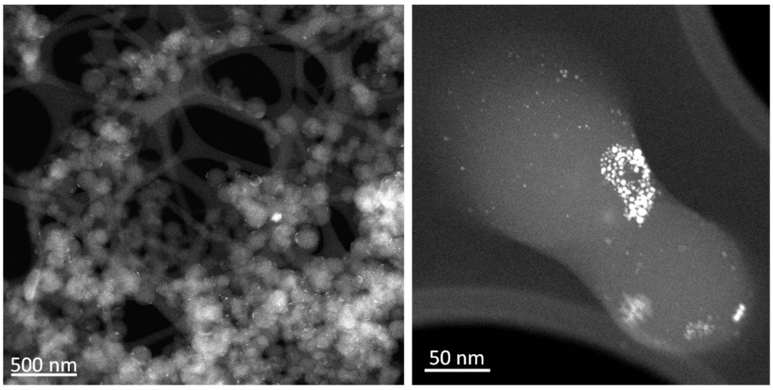

Figure S3. HAADF-STEM images of **AuPd PLGA** at different magnifications.

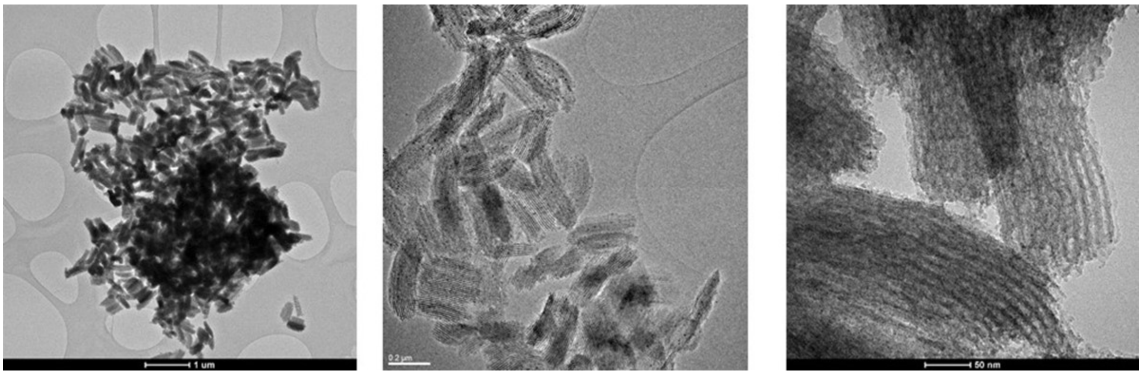

Figure S4. TEM images of the **AuPd** NPs embedded in mesoporous **SiO_2** nanorods at different magnifications.

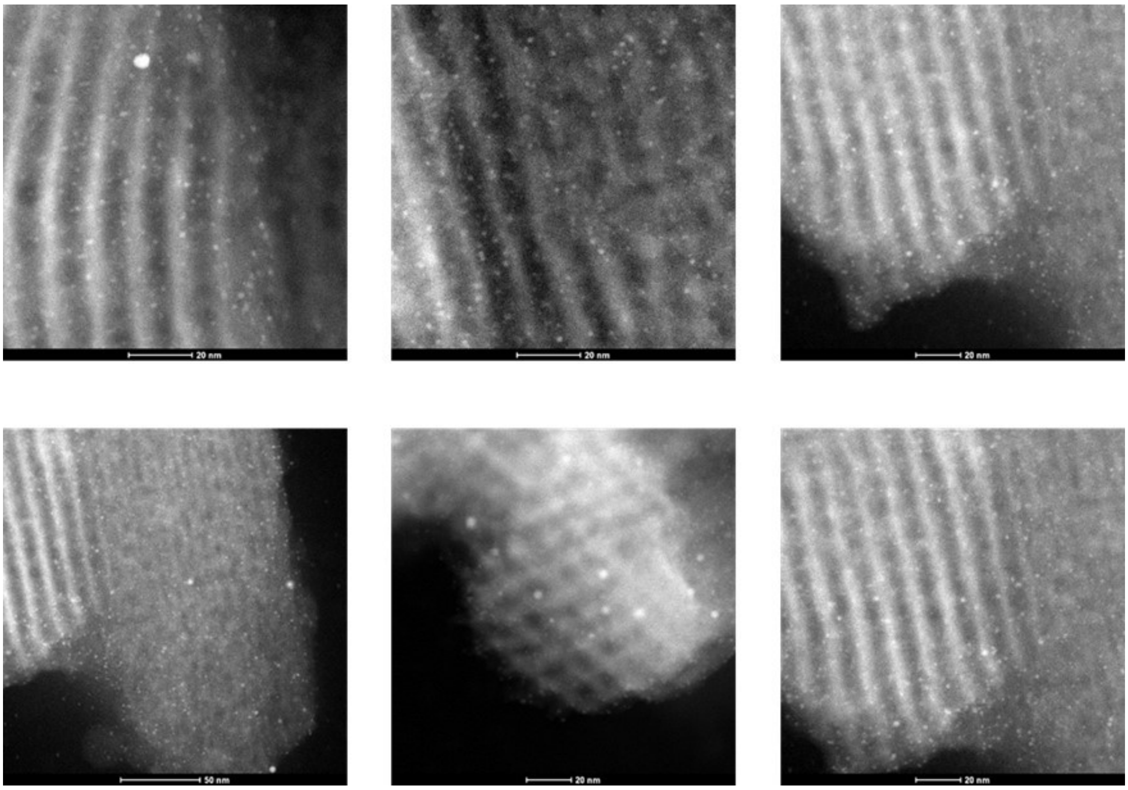

Figure S5. HAADF-STEM images of the **AuPd SiO_2** nanorods.

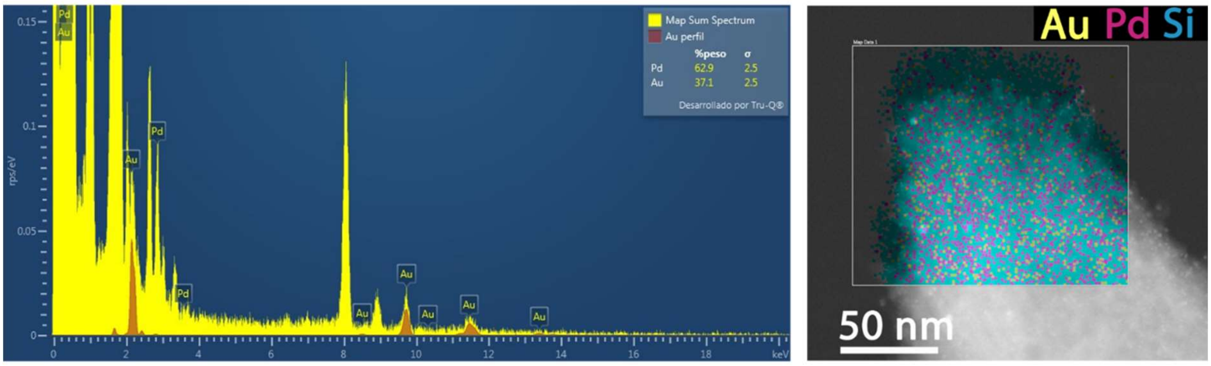

Figure S6. EDX spectrum and elemental mapping analysis of an **AuPd SiO_2**.

5. Biological Studies

5.1. Cell culture. Human lung adenocarcinoma A549 cells (a kind gift from Dr Simon Wilkinson) were cultured in Dulbecco's Modified Eagle Media (DMEM) supplemented with serum (10 % FBS) and L-glutamine (2 mM) and incubated in a tissue culture incubator at 37 °C and 5% CO_2.

5.2. Tolerability study. A549 cells were seeded in a 96-well plate (at 1,500 cells / well) and incubated for 24 h before treatment. Each well was then replaced with fresh media containing **Pd**, **PdPt**, **PdRu** and **AuPd NPs** at different concentrations (5, 10, 20 µg metal / mL) and incubated for 5 d. Untreated cells were used as control. Experiments were performed in triplicates. PrestoBlue™ cell viability reagent (10 % v/v) was added to each well and the plate incubated for 90 min. Fluorescence emission was detected using a PerkinElmer Victor multilabel reader (excitation filter at 540nm and emissions filter at 590nm). All conditions were normalized to the untreated cells (100%).

5.3. Determination of AuPd PLGA and AuPd SiO_2 cellular uptake by TEM. A549 cells were seeded in a 6-well plate format (at 400,000 cells / well) and incubated for 48 h. Each well was then replaced with a suspension of **AuPd PLGA** or **AuPd SiO_2** in culture media without serum (final concentration= 20 µg metal / mL). Untreated cells were used as control. After 30 min of incubation with **AuPd PLGA** or **AuPd SiO_2**, cells were washed twice with PBS, then detached with trypsin / EDTA and washed twice by centrifugation at 10,000 rpm for 5 min. Cells were fixed in 3% glutaraldehyde in 0.1 M sodium cacodylate buffer (pH 7.3) for 3 h then washed twice for 10 min with of 0.1 M sodium cacodylate buffer. Specimens were then post-fixed in 1% osmium tetroxide in 0.1 M sodium cacodylate for 45 min and then washed thrice for 10 min with of 0.1 M sodium cacodylate buffer. These samples were then dehydrated in 50 %, 70 %, 90 % and 100 % ethanol (x3) for 15 min each, then in two 10-min changes in propylene oxide. Samples were then embedded in TAAB 812 resin. Sections of 1 µm thick were cut on a Leica Ultracut ultramicrotome, stained with toluidine blue, and viewed in a standard microscope to select suitable areas for investigation. Ultrathin sections (60 nm thick) were cut from selected areas, stained in uranyl acetate and lead citrate and visualized using a JEOL JEM-1400 Plus TEM. Representative images were collected on a sCMOS direct detection camera.

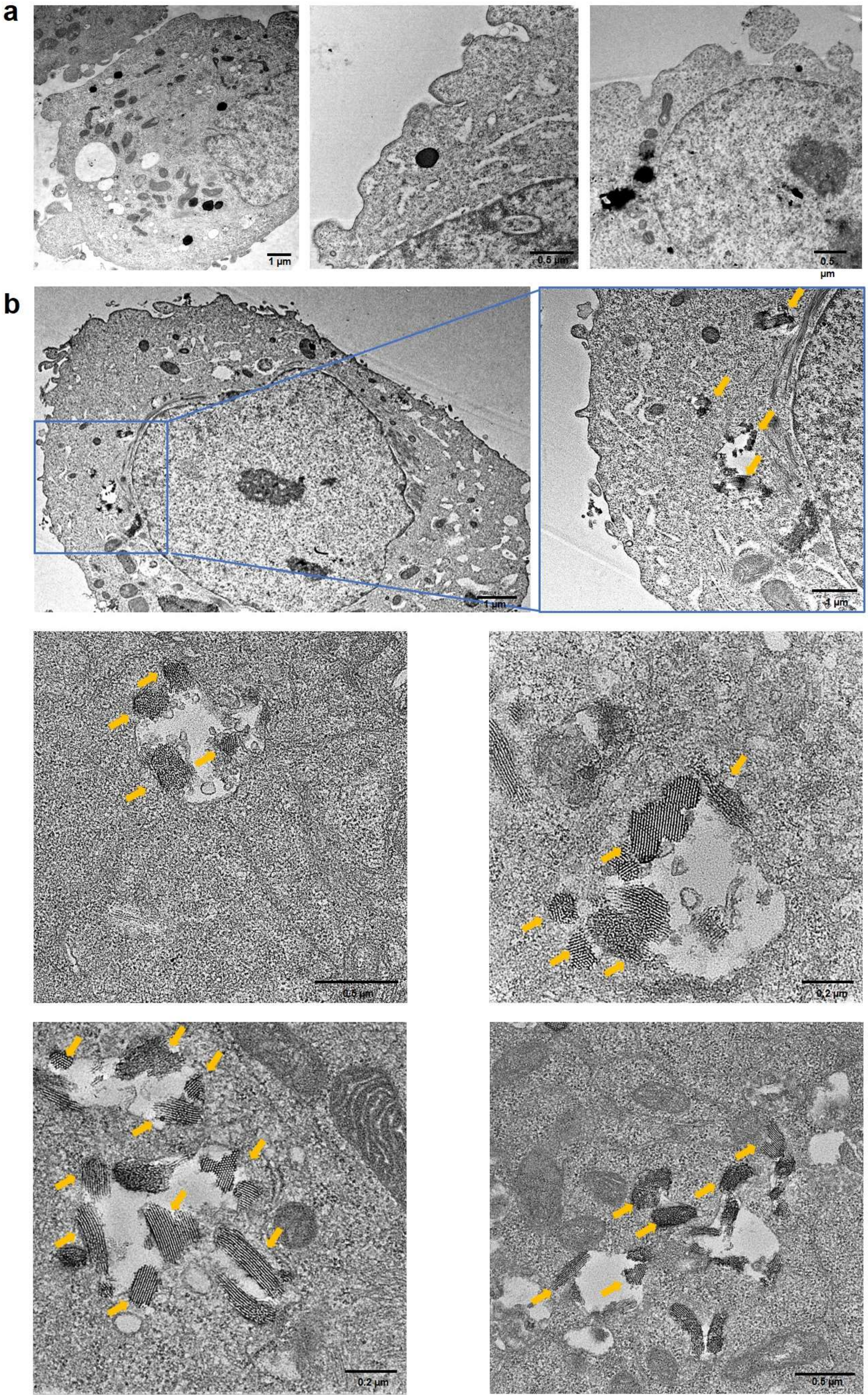

Figure S7. Nanoalloy internalization studies in lung cancer A549 cells. (a) Representative TEM images of ultrathin cross-section of cells treated with **AuPd PLGA**. (b) Representative TEM images of ultrathin cross-section of cells treated with **AuPd SiO_2**.

5.4. Determination of AuPd PLGA and AuPd SiO_2 cellular uptake by ICP-OES. A549 cells were seeded in a 96 well plate at 1500 cells / well and incubated for 24 h before treatment. **AuPd PLGA** and **AuPd SiO_2** (20 µg metal / mL) were added to each well and incubated for 6 h in culture media without serum. Then, media was removed and cells were washed twice with PBS. Trypsin (30 µL) was added in order to detach cells and neutralized using 10% FBS in PBS (30 µL). Cells were collected and centrifuged at 13,000 rpm for 5 min. Supernatant was discarded and cells were counted in a Neubauer chamber and digested in a 10% HNO_3 aqueous solution (200 µL) at 80°C overnight. The digested solution was diluted 5 times in distilled water and injected in an inductively coupled plasma-atomic emission spectrometer (Perkin Elmer Optima 5300 DV ICP-MS).

5.5. *In cellulo* Pro-PTX activation. A549 cells were plated at 1,500 cells / well in a 96-well plate and incubated for 24 h before treatment. The corresponding wells were then replaced with a suspension of **AuPd** NPs, **AuPd PLGA** or **AuPd SiO_2** (20 µg metal / mL) in DMEM media supplemented with L-glutamine. After 30 min of incubation, the media containing the NPs was removed, adhered cells were washed twice with PBS buffer, and subsequently treated with completed fresh media containing either DMSO (0.1% v/v) or **Pro-PTX** (1 µM). Cells treated with **PTX** and **Pro-PTX** (1 µM) were used as positive and negative controls, respectively. After treatment for 5 d, cell viability was determined as described above. The experiments were performed in triplicates.

5.6. Immunofluorescence study. To validate the antiproliferative mode of action of the bioorthogonally activated prodrug, we studied microtubules by immunofluorescence as follows. A549 cells were seeded on 10 mm poly(l-lysine) precoated coverslips in 24 well plates (5,000 cells / well). After 24 h, cells were incubated with/out **AuPd PLGA** or **AuPd SiO_2** for 30 min in serum-free media. The media was removed and cells washed twice with PBS. Then, **Pro-PTX** or **PTX** (1 µM) in 500 µL of DMEM was added to the cells in the presence or absence of **AuPd** (negative controls). After 48 h, cells were fixed with paraformaldehyde (4% v/v) for 10 min and washed 3 times with PBS every 5 min. Cells were permeabilized for 15 min with 0.3% Tween/PBS and washed three times with PBS every 5 min. Coverslips were incubated in blocking buffer (1X PBS, 5% Goat serum, 0.3% Triton X-100) for 60 min. Anti-α-tubulin Rabbit mAb (Cell Signaling Technology) was incubated overnight at 4°C in antibody dilution buffer (1X PBS, 1% BSA, 0.3% Triton X-100) at a dilution of 1:25. After washing three times with PBS, coverslips were incubated for 30 min in antibody dilution buffer (1X PBS, 1% BSA, 0.3% Triton X-100) with Alexa Fluor 488 Goat anti-Rabbit IgG (H+L) secondary antibody (Invitrogen) at a dilution of 1:400. Coverslips were washed three times with PBS and mounted on a slide with a 1:1 mixture of VECTASHIELD® HardsetTM Antifade Mounting Medium with Phalloidin: VECTASHIELD® Antifade Mounting Medium with DAPI. Cells were imaged using

a scanning confocal inverted microscope Olympus FluoView FV1000 with a 20x objective. The images were acquired using the FV10-ASW program in a sequential mode with software pre-configured settings for Alexa Fluor 488 and TIRTC and analyzed with Image-J software to obtain maximal projections and merged to obtain the composite images.

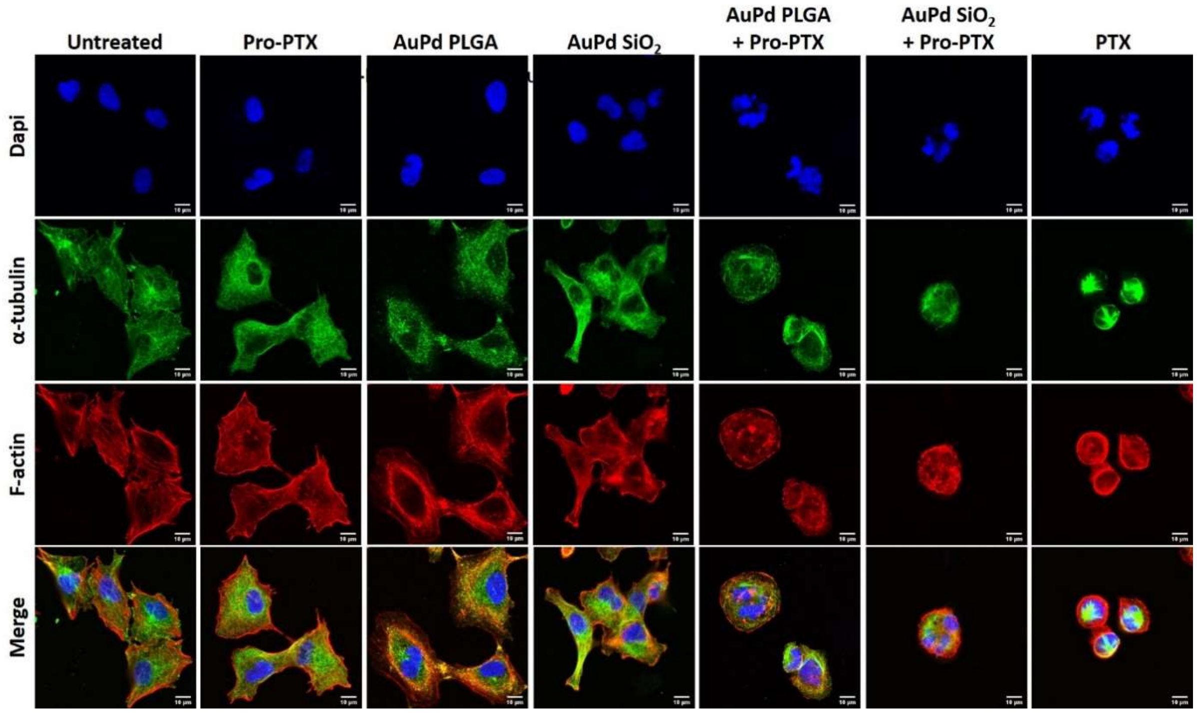

Figure S8. Immunofluorescence study. Experiments: DMSO; **Pro-PTX** (1 µM); **AuPd PLGA** or **AuPd SiO_2** (20 µg metal / mL); **Pro-PTX** (1 µM) + **AuPd PLGA** or **AuPd SiO_2** (20 µg metal / mL); **PTX** (1 µM). Cells were treated with encapsulated **AuPd** for 30 min prior to prodrug addition. After 48h treatment, cells were fixed and stained with DAPI (blue), anti-α-tubulin mAb (green) and phalloidin (red). Z-stack images are shown as maximal projections. Scale bar = 10 µm.

6. References

1. Weiss, J. T.; Dawson, J. C.; Macleod, K. G.; Rybski, W.; Fraser, C.; Torres-Sánchez, C.; Patton, E. E.; Bradley, M.; Carragher, N. O.; Unciti-Broceta, A.Extracellular palladium-catalysed dealkylation of 5-fluoro-1-propargyl-uracil as a bioorthogonally activated prodrug approach *Nat. Commun.* **2014**, 5, 3277.
2. Pérez-López, A.M.; Rubio-Ruiz, B.; Valero, T.; Contreras-Montoya, R.; Álvarez de Cienfuegos, L.; Sebastián, V.; Santamaría, J.; Unciti-Broceta, A. Bioorthogonal activation of paclitaxel by hydrogel-entrapped ultrathin Palladium nanosheets. *J. Med. Chem.* **2020,** *63*, 9650-9659.
3. Hueso, J. L.; Sebastian, V.; Mayoral, A.; Usón, L.; Arruebo, M.; Santamaría, J. Beyond gold: rediscovering tetrakis-(hydroxymethyl)-phosphonium chloride (THPC) as an effective agent for the synthesis of ultra-small noble metal nanoparticles and Pt-containing nanoalloys. *RSC Adv.* **2013**, *3*, 10427–10433.
4. Uson, L.; Sebastian, V.; Mayoral, A.; Hueso, J. L.; Eguiza-bal, A.; Arruebo M.; Santamaria, J. Spontaneous formation of Au–Pt alloyed nanoparticles using pure nano-counterparts as starters: a ligand and size dependent pro-cess. *Nanoscale*, **2015**, *7*, 10152–10161.
5. Uson, L.; Arruebo M.; Sebastian, V. Towards the continuous production of Pt-based heterogeneous catalysts using microfluidic systems. *Dalton Trans.* **2018**, *47*, 1693–1702.
6. Uson, L.; Yus, C.; Mendoza, G.; Leroy, E.; Irusta, S.; Alejo, T.; Garcia-Domingo, D.; Larrea, A.; Arruebo, M.; Arenal, R.; Sebastian, V. Nanoengineering palladium plasmonic nanosheets inside polymer nanospheres for photothermal therapy and targeted drug delivery. *Adv. Funct. Mater.* **2022**, *32*, 2106932.
7. Uson, L.; Hueso, J. L.; Sebastian, V.; Arenal, R.; Florea, I.; S. Irusta, S.; Arruebo, M.; Santamaria, J. In-situ preparation of ultra-small Pt nanoparticles within rod-shaped mesopo-rous silica particles: 3-D tomography and catalytic oxidation of n-hexane. *Catal. Commun.* **2017**, *100*, 93-97.
8. Ortega-Liebana, M.C.; Hueso, J.L.; Fernández-Pacheco, R.; Irusta, S.; Santamaria, J. Luminescent mesoporous nanorods as photocatalytic enzyme-like peroxidase surrogates. *Chem. Sci.* **2018**, *9*, 7766-7778.
9. Ortega-Liebana, M.C.; Chung, N.X.; Limpens, R.; Gomez, L.; Hueso, J.L.; Santamaria, J.; Gregorkiewiczc, T. Uniform luminescent carbon nanodots prepared by rapid pyrolysis of organic precursors confined within nanoporous templating structures. *Carbon* **2017**, *117*, 437-446.
